# Vitamin D receptor gene polymorphisms and haplotypes and postmenopausal breast cancer risk

**DOI:** 10.1186/bcr1994

**Published:** 2008-04-17

**Authors:** Sascha Abbas, Alexandra Nieters, Jakob Linseisen, Tracy Slanger, Silke Kropp, Elke Jonny Mutschelknauss, Dieter Flesch-Janys, Jenny Chang-Claude

**Affiliations:** 1 Division of Cancer Epidemiology, German Cancer Research Center, Im Neuenheimer Feld, 69120 Heidelberg, Germany; 2 Institute for Medical Biometrics and Epidemiology, University Clinic Hamburg-Eppendorf, Martinistr., 20246 Hamburg, Germany

## Abstract

**Introduction:**

Vitamin D receptor (VDR) genotypes may influence breast cancer risk by altering potential anticarcinogenic effects of vitamin D, but epidemiological studies have been inconsistent. Effect modification by serum 25-hydroxyvitamin D (25 [OH]D), the biomarker for vitamin D status in humans, has rarely been examined.

**Methods:**

We assessed the effects of two frequently analyzed polymorphisms (FokI and TaqI) and two potentially functional variants (*VDR*-5132 and Cdx2) in the *VDR *gene, which thus far have not been analyzed with respect to breast cancer risk, on postmenopausal breast cancer risk in a population-based, case-control study including 1,408 patients (cases) and 2,612 control individuals (controls) matched for year of birth. Odds ratios (ORs) for breast cancer adjusted for potential confounders were calculated for genotypes and estimated haplotypes.

**Results:**

No differences in serum 25(OD)D concentrations by *VDR *genotype were observed. None of the analyzed polymorphisms was associated with overall risk for postmenopausal breast cancer. However, the TaqI polymorphism was associated with a significantly increased risk for oestrogen receptor positive tumours (OR = 1.18, 95% confidence interval [CI] = 1.00 to 1.38, comparing t allele carriers with noncarriers) but not for oestrogen receptor negative tumours (OR = 0.88, 95% CI = 0.69 to 1.13; *P *for interaction = 0.04). Haplotype analysis revealed the haplotype FtCA (FokI F, TaqI t, *VDR*-5132 C, Cdx2 A), which contains the TaqI t allele, to be associated with a significantly greater breast cancer risk as compared with the most frequent haplotype FTCG (OR = 1.43, 95% CI = 1.00 to 2.05). No significant interaction between *VDR *genotypes or haplotypes and 25(OH)D was observed.

**Conclusion:**

Our results support potential effects of *VDR *polymorphisms on postmenopausal breast cancer risk and possible differential effects of receptor status of the tumour. However, further studies focusing on the influence of polymorphisms and haplotypes on VDR functionality, activity and concentration are needed.

## Introduction

In various observational studies vitamin D intake and serum concentrations of vitamin D metabolites have been associated with decreased risk for developing breast cancer [1-3]. Apart from the role that vitamin D plays in maintaining calcium homeostasis, its antiproliferative effects – by influencing cell differentiation, cell growth and apoptosis – are well established [4-6].

Vitamin D from both diet and endogenous production is converted via two consecutive hydroxylation steps to 25-hydroxyvitamin D (25 [OH]D) and to 1,25-dihydroxyvitamin D (1,25 [OH]_2_D). The biologically most active form of vitamin D is 1,25(OH)_2_D, which mainly exerts its antiproliferative effects by binding to the vitamin D receptor (VDR) and acting in complex as a transcriptional factor for a variety of genes, including those involved in cell differentiation and cell growth [7]. The VDR is present in a variety of cell types, including malignant and normal breast cells [8,9]. Various studies have assessed associations between various polymorphisms in the *VDR *gene and breast cancer risk, with inconsistent results. These polymorphisms include three frequently analyzed, highly linked single nucleotide polymorphisms (SNPs) BsmI, ApaI and TaqI at the 3' end of the *VDR *gene. The t allele of the TaqI SNP in exon 9 (rs731236, T/C, C = t), which leads to a silent codon change, has been found in different studies to be associated with a nonsignificantly increased breast cancer risk [10] and with a decreased risk for breast cancer [11], or there was no association at all [12-17].

Another promising functional polymorphism in the start codon at the 5' promotor region of the *VDR *is the FokI SNP (rs2228570/rs10735810, T/C, T = f). The f allele leads to a protein that is three amino acids longer and less effective [18,19] and was associated with a statistically significant increased breast cancer risk in a case-control study nested within the Nurses Health Study (NHS) [20]. However, other studies did not find any association [11,14,21-23]. Furthermore, two potentially functional polymorphisms [24-26] located in two transcription factor binding sites within the *VDR *promoter region have been reported: *VDR*-5132 (rs1989969, T/C), which has been related to a potential elimination of a GATA-1 transcription factor binding site [25]; and Cdx2 (rs11568820, G/A), which leads to decreased transcriptional activity of the *VDR *promoter [26]. The Cdx2 polymorphism has been associated with risk for bone fracture [27,28] and with risk for prostate cancer in 25(OH)D deficient men [29]; the *VDR*-5132 polymorphism has been related to risk for prostate cancer [25]. To our knowledge, neither polymorphism has yet been examined with respect to breast cancer risk.

The majority of studies assessing polymorphisms in the *VDR *gene and breast cancer risk have been very small and have often failed to account for known breast cancer risk factors and potential confounders in their analyses. Only one study has thus far assessed the association of breast cancer risk and *VDR *gene polymorphisms in relation to serum 25(OH)D [20], and it found no significant interactions. We recently reported an inverse association between serum 25(OH)D concentration and postmenopausal breast cancer risk in a large German case-control study [30]. We therefore assessed the association of FokI, TaqI, *VDR*-5132 and Cdx2 SNPs and their associated haplotypes with postmenopausal breast cancer risk and possible effect modification by serum 25(OH)D in this study population.

## Materials and methods

### Study population and data collection

We conducted a large population-based, case-control study (MARIE [Mamma Carcinoma Risk factor Investigation] study) that was conducted in two regions in Germany: the city of Hamburg and the Rhein-Neckar-Karlsruhe (R-N-K) region. The study was approved by the ethics committees of both the University of Heidelberg and the University of Hamburg, and it was conducted in accordance with the Declaration of Helsinki. All study participants gave informed consent. Patients (cases) were eligible if they had a histologically confirmed primary invasive or *in situ *breast cancer diagnosed between 1 January 2001 and 30 September 2005 in Hamburg and between 1 August 2002 and 31 July 2005 in the R-N-K region. Further inclusion criteria were age between 50 and 74 years and being a resident of one of the study regions. Cases were identified through frequent monitoring of hospital admissions, surgery schedules and pathology records. Clinical and pathological characteristics of the patients were abstracted from hospital and pathology records. Of the 5,970 eligible patients who could be contacted, 3,919 (65.6%) participated and 2,051 (34.4%) declined to participate or did not respond to letters of invitation.

Two control individuals (controls) per case were randomly selected from lists of residents provided by population registries and frequency matched by year of birth and study region to the cases. Of the 17,093 controls who met the inclusion criteria, 7,421 (43.4%) participated, 7,521 (44.0%) refused to participate and 2,151 (12.6%) did not respond.

Using a standardized questionnaire all participants were interviewed by trained personnel to obtain information on sociodemographic factors and potential breast cancer risk factors.

Women who reported their last natural menstrual bleeding at least 12 months before the reference date (age at diagnosis and recruitment for cases and controls, respectively), a bilateral oophorectomy, or cessation of menses because of radiation or chemotherapy were defined as postmenopausal. Women older than 55 years with unclear menopausal status because of hysterectomy or hormone use were also considered postmenopausal (because the 90th percentile for age at menopause in women with natural menopause was 55 years). Premenopausal women and women under age 55 years with unclear menopausal status were excluded from the analysis. In total, 3,464 invasive or *in situ *breast cancer cases and 6,657 controls were classified as postmenopausal, of whom 1,559 cases and 3,008 controls were from the R-N-K region. Because of organizational aspects of sample handling, we included only participants from the R-N-K study region.

### Genotyping analysis

Postmenopausal participants from the R-N-K region with DNA samples were included in this analysis, which included 1,408 (90.3%) cases and 2,612 (86.8%) controls.

Genomic DNA was extracted from blood samples using FlexiGene DNA Kit (Qiagen, Hilden, Germany), in accordance with the manufacturer's instruction. The TaqI SNP was genotyped by PCR-restriction fragment length polymorphism. After PCR reaction, 5 μl of the PCR product was digested with 1 unit of *TaqI *restriction enzyme (New England Biolabs, Ipswich, MA, USA). The resultant fragments (242 base pairs [TT genotype], 131 and 111 base pairs [CC], and 111, 131 and 242 base pairs [TC]) were resolved on a 3.2% agarose gel. The FokI, *VDR*-5132 and Cdx2 polymorphisms were genotyped using Pyrosequencing™ technology (Biotage, Uppsala, Sweden) [31]. PCR mixtures contained 5 ng DNA, 1× Ready Mix PCR buffer (ABgene, Epsom, UK), 0.25 U Thermoprime DNA polymerase (ABgene), deoxynucleoside triphosphates (each at 167 μmol/l; PeqLab, Erlangen, Germany) and primers (3 pmol each), in a total reaction volume of 12 μl. Cycling conditions were identical for all SNPs, namely 40 cycles (except for Cdx2 [45 cycles]) of 94°C for 40 seconds, 57°C for 40 seconds and 72°C for 40 seconds.

The following primers (5' → 3') were used: TaqI: forward CTGCCGTTGAGTGTCTGTGT and reverse TCGGCTAGCTTCTGGATCAT; FokI: AGGGCGAATCATGTATGAGG (PCR), GGTCAAAGTCTCCAGGGTCA (biotinylated) and TTGCTGTTCTTACAGGG (sequencing); *VDR*-5132: TGTCCTCATTTGGCCCCAGGA (PCR), ACCGGGTGGATGCAGAAAGG (bio) and GGGTGGTTGTCTA (seq); and Cdx2: CCCAAAAGGAAAGGAAGGAA (PCR), AAAGCAAACCAAGGGGTCTT (bio) and CCTGAGTAAACTAGGTCACA (seq). For quality control, 10% of the samples were selected at random for repeated genotyping and concordance was 100%. Samples with ambiguous results were repeated. The overall success rate for genotyping was above 99% for all four polymorphisms analyzed.

### Measurement of 25-hydroxyvitamin D

For quantification of 25(OH)D in serum, we used the OCTEIA 25-hydroxyvitamin D enzyme immunoassay (IDS, Immunodiagnostic Systems Limited, Boldon, UK). We analyzed a subset of 1,391 postmenopausal cases and 1,365 randomly selected postmenopausal controls from the R-N-K region matched for year of birth (continuous) and time of blood collection (January to March, April to June, July to September, or October to December) in a single batch between November 2006 and January 2007. The coefficient of variation was 3.4% for intra-assay determination and 7.6% for inter-assay determination. We measured 235 random samples (8.5%) in duplicate. The average absolute deviation from the mean between two duplicates was 2.2%.

### Data analysis

Polymorphisms were tested for deviation from Hardy-Weinberg equilibrium (HWE) by comparing the observed and expected genotype frequencies using the χ^2 ^test. To estimate linkage disequilibrium for each pair of polymorphisms, we calculated r^2^.

Women were categorized both by genotype (homozygote minor allele, heterozygote, or homozygote major allele) and by carrier status (homozygote major allele or carrier of the minor allele) for the respective polymorphisms. We assessed the association of SNPs in the *VDR *gene and postmenopausal breast cancer risk by means of conditional logistic regression with stratification by year of birth (continuous) and additional stratification by time of blood collection (January to March, April to June, July to September, or October to December) in models assessing interaction with 25(OH)D. Homozygote major allele carriers were used as the reference category. We present odds ratios (ORs) and their corresponding 95% confidence intervals (CIs) under the assumption of a general and a dominant inheritance model. The following breast cancer risk factor variables were included in the multivariate model: age at menopause (< 47 years, 47 to 51 years, 52 to 55 years, ≥56 years, or unknown), body mass index (< 22.5 kg/m^2^, 22.5 to < 25 kg/m^2^, 25 to < 30 kg/m^2^, or ≥30 kg/m^2^), education level (low, middle, or high), first-degree family history of breast cancer (yes, no, or unknown), history of benign breast disease (yes or no), number of pregnancies (to week 28 or beyond; 0, 1, 2, ≥3), age at menarche (< 12 years, 12 to 14 years, of ≥15 years), breast feeding history (ever or never), total number of mammograms (0, 1 to 4, 5 to 9, ≥10, or unknown), smoking status (never, past, or current) and use of menopausal hormone therapy (never, past, or current).

Haplotype analysis was conducted using the R haplo.stats package [32]. Haplotypes and haplotype frequencies were estimated using the R function haplo.em. The association of common haplotypes with breast cancer risk was evaluated with the R function haplo.glm. Haplo.glm applies a haplotype-trait association test based on a general linear model framework using maximum likelihood estimates for haplotype effects, allowing for ambiguity of haplotype phase [33]. A log-additive risk model was assumed, in which haplotype specific regression coefficients represent the change in the log odds of disease for every additional copy of the haplotype compared with the homozygote reference haplotype. Because haplo.glm uses an unconditional approach, we adjusted for the same covariates as in the genotype analysis model but additionally adjusted for the matching variable year of birth and for time of blood collection in models assessing interaction with 25(OH)D. The most common haplotype was set as the reference haplotype.

Statistical genotype-environment interaction was evaluated with the likelihood ratio test by including a cross-product term of the dichotomous *VDR *genotype variable (carriers versus noncarriers) and potential interaction variables of interest (continuous variable for 25 [OH]D) in the multivariate model. Statistical haplotype-environment interaction was evaluated using Wald statistics for the respective interaction term in haplo.glm, including interaction terms for all haplotypes simultaneously in the model.

Interaction of genotypes or haplotypes with oestrogen receptor (ER) or progesterone receptor (PR) status of the tumour was assessed by means of a case-only analysis. ER or PR status was used as the dependent variable (outcome) and potential interaction variables (genotypes and haplotypes) as independent variable in the logistic regression model. The Wald statistic for the respective independent variable was used to test for statistical interaction.

All tests were two-sided and considered to be statistically significant at *P *≤ 0.05. All calculations except the haplotype analysis were conducted using the statistical software SAS 9.1 (SAS Institute Inc., Cary, NC, USA).

## Results

In comparison with controls, and consistent with established risk factors for breast cancer, cases were significantly older at menopause, were more likely to have a positive family history of breast cancer, more frequently had a history of benign breast disease, had lower parity, were younger at menarche, were less likely to have breastfed, had a greater number of previous mammograms, and more frequently used hormone therapy (Table 1).

**Table 1:** Characteristics and risk factors for postmenopausal breast cancer in cases and matched controls in the study population

Characteristics	Cases (n^a ^= 1,408)		Controls (n^a+ ^= 2,612)		*P*^b^
	n	%	n	%	
Age at diagnosis/recruitment (years)					0.87
50 to 54	104	7.4	191	7.3	
55 to 59	301	21.4	528	20.2	
60 to 64	447	31.7	859	32.9	
65 to 69	380	27.0	720	27.6	
≥70	176	12.5	314	12.0	
BMI (kg/m^2^)					0.68
< 22.5	554	39.4	1,043	39.9	
22.5 to < 25	473	33.6	829	31.8	
25 to < 30	323	22.9	624	23.9	
≥30	58	4.1	114	4.4	
Educational level					0.07
Low	912	64.8	1,761	67.4	
Middle	305	21.6	559	21.4	
High	191	13.6	292	11.2	
Age at menopause (years)					< 0.01
< 47	146	10.4	396	15.2	
47 to 51	407	28.9	748	28.6	
52 to 55	248	17.6	402	15.4	
≥56	63	4.5	115	4.4	
Unknown	544	38.6	951	36.4	
First degree family history of breast cancer					< 0.01
No	1,113	79.1	2,164	82.8	
Yes	234	16.6	329	12.6	
Unknown	61	4.3	119	4.6	
Benign breast disease					< 0.01
No	860	61.3	1,833	70.5	
Yes	544	38.7	766	29.5	
Number of pregnancies (≥ 28^th ^week)					< 0.01
0	200	14.2	294	11.3	
1	383	27.2	635	24.3	
2	518	36.8	1,031	39.5	
≥3	307	21.8	652	24.9	
Age at menarche (years)					0.02
< 12	125	8.9	221	8.5	
12 to 14	950	67.5	1,658	63.7	
≥15	333	23.6	725	27.8	
Ever breastfeeding					< 0.01
No	555	39.4	868	33.2	
Yes	853	60.6	1,744	66.8	
Number of mammograms in total					< 0.01
0	195	13.8	334	12.8	
1 to 4	598	42.5	1,370	52.5	
5 to 9	343	24.4	599	22.9	
≥10	254	18.0	288	11.0	
Unknown number	18	1.3	21	0.8	
Use of hormone therapy					< 0.01
Never	528	37.9	1,113	43.1	
Past	293	21.0	672	26.0	
Current (≤ 6 months)	572	41.1	798	30.9	
Hormonal receptor status of the tumor^c^					
ER-positive	994	76.3			
ER-negative	308	23.7			
PR-positive	848	65.2			
PR-negative	453	34.8			
Time of blood collection^d^					0.99
January to March	317	22.8	311	22.8	
April to June	292	21.0	286	21.0	
July to September	402	28.9	392	28.7	
October to December	380	27.3	376	27.5	

^a^Numbers do not always add up to total numbers because of missing values. ^b^χ^2 ^test for differences between cases and controls. ^c^Data on estrogen receptor (ER) and progesterone receptor (PR) status was available for 1,302 and 1,301 invasive tumour cases (*in situ *tumours excluded), respectively. ^d^Data on serum 25-hydroxyvitamin D status were available for 1,391 cases and 1,365 controls. BMI, body mass index.

Median 25(OH)D concentrations were 44.9 nmol/l and 51.5 nmol/l for cases and controls, respectively. We did not observe significant differences in 25(OH)D concentrations by genotype in any of the four analyzed polymorphisms (Table 2).

**Table 2:** Serum 25(OH)D concentrations in the control group by polymorphisms in the vitamin D receptor gene

Polymorphism	Genotype			*P*^a^
TaqI	TT: 49.8 (32.6–67.5)	Tt: 47.1 (32.5–66.1)	tt: 48.1 (32.7–69.8)	0.52
FokI	FF: 49.2 (33.0–66.9)	Ff: 48.2 (32.4–67.4)	ff: 47.9 (31.7–69.7)	0.85
*VDR*-5132	CC: 49.1 (32.9–66.9)	CT: 48.6 (32.6–68.3)	TT: 47.7 (31.6–66.9)	0.78
Cdx2	GG: 48.5 (32.5–67.4)	GA: 48.7 (31.9–66.2)	AA: 50.7 (35.0–68.6)	0.56

Values are expressed as median serum 25-hydroxyvitamin D (25 [OH]D; 25th to 75th percentile) in nmol/l. ^a^Wilcoxon rank-sum test for median differences by genotype.

The genotype distribution in the control group was in HWE for all analyzed polymorphisms (*P *= 0.30, *P *= 0.31, *P *= 0.39 and *P *= 0.16 for FokI, TaqI, *VDR*-5132 and Cdx2, respectively). The observed allele frequencies in the controls were comparable to those reported in the dbSNP database for Caucasian populations (minor allele frequencies of 0.39, 0.39, 0.41 and 0.19 for FokI, TaqI, *VDR*-5132 and Cdx2, respectively, in our study population). There was no evidence of linkage disequilibrium between any pair of the four analyzed polymorphism (r^2 ^< 0.01).

We did not observe a significant association between genotypes of any of the four analyzed polymorphisms and risk for postmenopausal breast cancer (Table 3). In the subpopulation for whom there were data on serum 25(OH)D (1,391 cases and 1,365 controls), we further examined whether the association between the *VDR *genotypes and breast cancer risk differed by serum 25(OH)D level. No departure from multiplicative interaction was observed when considering serum 25(OH)D level as a continuous variable (*P *for interaction = 0.39, 0.43, 0.51 and 0.61 for FokI, TaqI, *VDR*-5132 and Cdx2, respectively). Differential effects for the respective polymorphisms were also not found when serum 25(OH)D concentrations were dichotomized at 30 nmol/l, which was defined as the cutt-off point for vitamin D deficiency. Analyses stratified by season did not yield any further significant 25(OH)D-genotype interactions or differences in results between the winter months (October to March) and the summer months (April to September). There was also no significant gene-environment interaction with family history of breast cancer, use of hormone therapy, or smoking status.

**Table 3:** Odds ratios for postmenopausal breast cancer by polymorphisms in the vitamin D receptor gene

Genotype	Cases		Controls		Crude model^a^		Adjusted model^b^		
	n	%	n	%	OR (95% CI)		OR (95% CI)		*P*^c^
TaqI	1,403		2,609						0.33
TT	497	35.4	980	37.6	1		1		
Tt	667	47.6	1,218	46.7	1.08	(0.94–1.25)	1.08	(0.93–1.26)	
tt	239	17.0	411	15.7	1.15	(0.95–1.39)	1.11	(0.91–1.36)	
Tt/tt					1.10	(0.96–1.26)	1.09	(0.95–1.26)	
FokI	1,390		2,596						0.24
FF	566	40.7	998	38.5	1		1		
Ff	606	43.6	1,203	46.3	0.88	(0.77–1.02)	0.89	(0.77–1.03)	
ff	218	15.7	395	15.2	0.98	(0.80–1.19)	0.97	(0.79–1.19)	
Ff/ff					0.91	(0.80–1.04)	0.91	(0.79–1.05)	
*VDR*-5132	1,400		2,607						0.92
CC	488	34.9	892	34.2	1		1		
CT	683	48.8	1,284	49.3	0.99	(0.85–1.14)	1.00	(0.86–1.16)	
TT	229	16.3	431	16.5	0.99	(0.81–1.20)	1.01	(0.83–1.24)	
CT/TT					0.99	(0.86–1.13)	1.00	(0.87–1.16)	
Cdx2	1,406		2,606						0.22
GG	888	63.1	1,701	65.3	1		1		
GA	465	33.1	795	30.5	1.11	(0.97–1.28)	1.08	(0.94–1.25)	
AA	53	3.8	110	4.2	0.94	(0.67–1.32)	0.94	(0.66–1.33)	
GA/AA					1.09	(0.95–1.25)	1.07	(0.93–1.23)	

^a^Conditional logistic regression stratified by year of birth. ^b^Conditional logistic regression stratified by year of birth adjusted for age at menopause, first-degree family history of breast cancer, history of benign breast disease, number of pregnancies (≥28th week), age at menarche, breastfeeding history, total number of mammograms, use of hormone therapy, body mass index, education level and smoking status. ^c^χ^2 ^test for difference between cases and controls comparing the three genotypes for each polymorphism. CI, confidence interval; OR, odds ratio.

Because vitamin D possibly exerts its anticarcinogenic activities via the oestrogen pathway, we assessed possible differential effects by receptor status of the tumour. We observed a statistically significant interaction for the TaqI polymorphism and ER status in a case-only model (*P *for interaction = 0.04; Table 4). Comparing t allele carriers with noncarriers, we found a statistically significantly increased OR of 1.18 (95% CI = 1.00 to 1.38) for ER-positive tumours, and an OR of 0.88 (95% CI = 0.69 to 1.13) for ER-negative tumours. No statistically significant interaction was observed between the TaqI polymorphism and PR status of the tumour, or between the FokI, Cdx2, or *VDR*-5132 polymorphism and PR or ER status (Table 4). We also did not observe any differences in main or interaction effects by stage of disease, when we performed analysis stratified by local versus advanced tumours, according to TNM-staging of the tumour (Union Internationale Contre le Cancer classification).

**Table 4:** Odds ratios for postmenopausal breast cancer by genotypes in the vitamin D receptor gene according to ER and PR status of the tumour

Genotype		ER-positive tumours		ER-negative tumours		PR-positive tumours		PR-negative tumours	
	n (controls)	n (cases)	OR (95% CI)	n (cases)	OR (95% CI)	n (cases)	OR (95% CI)	n (cases)	OR (95% CI)
TaqI									
TT	980	337	1	121	1	297	1	159	1
Tt/tt	1,629	653	1.18 (1.00–1.38)	186	0.88 (0.69–1.13)	547	1.10 (0.93–1.30)	293	1.11 (0.89–1.37)
FokI									
FF	998	394	1	127	1	331	1	190	1
Ff/ff	1,598	586	0.95 (0.81–1.10)	177	0.86 (0.67–1.10)	505	0.96 (0.82–1.14)	257	0.84 (0.68–1.03)
VDR-5132									
CC	892	347	1	113	1	298	1	162	1
CT/TT	1,715	640	0.99 (0.84–1.16)	194	0.92 (0.71–1.18)	543	0.97 (0.82–1.15)	290	0.97 (0.78–1.20)
Cdx2									
GG	1,701	641	1	190	1	546	1	283	1
GA/AA	905	351	1.01 (0.86–1.17)	118	1.14 (0.88–1.46)	301	1.01 (0.86–1.20)	169	1.11 (0.90–1.38)

We conducted a conditional logistic regression stratified by year of birth adjusted for age at menopause, first-degree family history of breast cancer, history of benign breast disease, number of pregnancies (≥28th week), age at menarche, breastfeeding history, total number of mammograms, use of hormone therapy, body mass index, education level and smoking status. Data on oestrogen receptor (ER) and progesterone receptor (PR) status was available for 1,302 and 1,301 cases, respectively.

We further estimated haplotypes by the expectation-maximization algorithm and included 15 haplotypes with a frequency above 1% in our analysis. The global test for an association of any haplotype and postmenopausal breast cancer risk was not significant (*P *global = 0.72). However, under the log-additive model, the haplotype FtCA (FokI F, TaqI t, *VDR*-5132 C and Cdx2 A) was significantly associated with breast cancer risk compared with the most common haplotype FTCG (OR = 1.43, 95% CI = 1.00 to 2.05; Table 5). Under the dominant model the OR was 1.53 (95% CI = 1.03 to 2.30) for those with at least one copy of the FtCA haplotype as compared with homozygote FTCG carriers. Power was inadequate using the recessive model. No statistical interaction was observed between any haplotypes and serum 25(OH)D concentration.

**Table 5:** Odds ratios for postmenopausal breast cancer by haplotypes in the vitamin D receptorgene under the log-additive model

Haplotype^a^	Haplotype frequencies (%)		OR (95% CI)			
	
	Cases (n = 1,408)	Controls (n = 2,612)	Crude model^b^		Adjusted model^c^	
FTCG	0.178	0.183	1		1	
FTCA	0.036	0.049	0.76	(0.51–1.15)	0.81	(0.53–1.23)
FTTG	0.124	0.116	1.12	(0.87–1.43)	1.09	(0.84–1.40)
FTTA	0.025	0.025	1.10	(0.69–1.77)	1.16	(0.71–1.88)
FtCG	0.117	0.113	1.07	(0.83–1.38)	1.06	(0.82–1.39)
FtCA	0.052	0.035	1.50	(1.07–2.12)	1.43	(1.00–2.05)
FtTG	0.076	0.079	0.98	(0.76–1.27)	1.04	(0.80–1.36)
FtTA	0.016	0.015	1.05	(0.59–1.87)	0.98	(0.54–1.79)
fTCG	0.109	0.117	0.97	(0.75–1.24)	0.97	(0.75–1.26)
fTCA	0.028	0.023	1.19	(0.72–1.96)	1.17	(0.70–1.95)
fTTG	0.080	0.083	0.98	(0.75–1.26)	1.04	(0.80–1.35)
fTTA	0.011	0.011	1.07	(0.48–2.37)	0.93	(0.41–2.09)
ftCG	0.048	0.041	1.19	(0.84–1.69)	1.19	(0.82–1.72)
ftCA	0.024	0.027	0.94	(0.57–1.55)	0.95	(0.56–1.64)
ftTG	0.065	0.072	0.94	(0.71–1.25)	0.94	(0.70–1.27)

^a^FokI, TaqI, VDR-5132, Cdx2 (with > 1% frequency in the study population). ^b^Unconditional haplotype analysis adjusted for matching factor year of birth. ^c^Unconditional haplotype analysis adjusted for year of birth, age at menopause, first-degree family history of breast cancer, history of benign breast disease, number of pregnancies (≥28th week), age at menarche, breastfeeding history, total number of mammograms, use of hormone therapy, body mass index, education level and smoking status. CI, confidence interval; OR, odds ratio.

Analogous to the genotype analysis, we evaluated statistical interaction of haplotypes with receptor status of the tumour in a case-only analysis. We did not observe an interaction of any haplotype with ER status. For the haplotype FtCA with the significant main effect, the ORs were 1.34 (95% CI = 0.90 to 2.01) and 1.67 (95% CI = 0.94 to 2.99) for ER-positive and ER-negative tumours, respectively (*P *for interaction = 0.64). A statistically significant interaction was found for the haplotype FtTA with PR status of the tumour (*P *for interaction = 0.03), but haplotype frequency was very low (*f*_FtTA _= 0.016).

In a sensitivity analysis of haplotype associations, we additionally adjusted for 25(OH)D concentration in the subpopulation for whom there were data on 25(OH)D. ORs did not change substantially, but the OR for the risk haplotype FtCA was no longer significant because of lower numbers of cases and controls.

## Discussion

In this population-based, case-control study, none of the polymorphisms in the *VDR *gene were associated with postmenopausal breast cancer risk. However, there was effect modification by ER status of the tumour, so that t allele carriage of the TaqI polymorphism was associated with significantly higher breast cancer risk as compared with noncarriage in ER-positive tumours only. Several studies reported no significant association between the TaqI polymorphism and breast cancer risk [10,12-17], and only one study comparing allele frequencies found a significantly higher risk for the T allele [11]. However, these studies did not have sufficient power to differentiate by receptor status of the tumour. Our findings of an effect of TaqI polymorphism only for ER-positive tumours suggest an oestrogen-mediated anticarcinogenic effect of vitamin D. Indeed, there are laboratory data that support the hypothesis that the anticarcinogenic effects of vitamin D could be mediated via the oestrogen pathway by downregulation of the ER and thus attenuating estrogenic bioresponses such as cell growth [34,35]. In addition, a putative vitamin D response element, serving as a binding site for the VDR-1,25(OH)_2_D-transcription factor complex, was found in the *ER *promoter [36].

The functionality of the TaqI polymorphism is unclear. Although TaqI is synonymous and in linkage disequilibrium with the two nonfunctional intron-located polymorphisms BsmI and ApaI, linkage disequilibrium extends into the 3' regulatory region, which is known to be involved in regulation of *VDR *expression [37]. Overall, functional studies – including studies on *VDR *mRNA expression – are inconsistent but tend to indicate a phenotype correlation with the frequently analyzed BAt haplotype (BsmI B ApaI A and TaqI t), including the TaqI t allele [37]. However, data for 41 individuals in whom *VDR *mRNA and protein level were measured indicated significantly lower levels in both mRNA and protein in those with the tt genotype [38]. This is consistent with our finding of higher breast cancer risk for the TaqI t allele in ER-positive tumours, because less *VDR *mRNA and protein may result in less 1,25(OH)_2_D-VDR complexes and therefore in less anticarcinogenic activity.

To our knowledge, this is the first study to examine Cdx2 and *VDR*-5132 polymorphisms and breast cancer risk. *VDR*-5132 leads to potential elimination of a GATA-1 transcription factor binding site [25], whereas Cdx2 leads to decreased transcriptional activity of the *VDR *promoter [26]. However, we found no association of Cdx2 or *VDR*-5132 SNPs with breast cancer risk in our study.

Our results for the FokI polymorphism do not support the findings of a large case-control study nested in the NHS reporting a significantly increased risk with the ff versus the FF genotype [20]. Although this polymorphism is known to be functional [18,39], numerous other studies, including the present one, were unable to confirm the finding from the NHS [11,14,21-23].

In a haplotype analysis, we found the haplotype FtCA (FokI F, TaqI t, *VDR*-5132 C, Cdx2 A) to be associated with a significantly greater breast cancer risk as compared with the most frequent haplotype (FTCG). The reason for this finding is unclear. The FtCA haplotype (versus the FTCG reference haplotype) contains the transcriptionally more active Cdx2 A allele [26], which is expected to be associated with a decreased risk for breast cancer. On the other hand, in accordance with the observed increased risk associated with TaqI t allele carriage in ER-positive tumours, the t allele is also present in the FtCA risk haplotype. Nevertheless, because of the multiple comparisons in our analysis, we cannot exclude the possibility that the observed significant associations may be chance findings.

We recently reported an inverse association between serum 25(OH)D concentration and postmenopausal breast cancer risk [30] and therefore were interested in assessing possible interaction between 25(OH)D status and genotype. Gene-environment interactions may explain inconsistencies in associations between polymorphisms and breast cancer risk in different studies. However, we did not find evidence for interactions of *VDR *polymorphisms with 25(OH)D (the observed inverse association of 25 [OH]D and breast cancer risk was not modified by genotype). Our results corroborate the findings in the NHS of an absence of interaction between the FokI SNP and 25(OH)D [20]. Functional variants of the *VDR *might affect 25(OH)D concentration, because the VDR is possibly involved in negative feedback regulation of 1,25(OH)_2_D synthesis mediated by the 1α-hydroxylase, which is the enzyme that converts 25(OH)D to active 1,25(OH)_2_D [40]. However, 25(OH)D levels did not vary by *VDR *genotype in our population, which is consistent with results on the relation between FokI and TaqI polymorphisms and vitamin D status in previous smaller studies [38,41].

Strengths of our study are the large sample size, the adjustment for all potential breast cancer risk factors, the evaluation of interactions with serum 25(OH)D, and the restriction to postmenopausal women, because various studies so far have not differentiated between premenopausal and postmenopausal women. Genotyping errors can almost completely be excluded, because 10% of the samples were genotyped in duplicate and concordance was 100%. In addition, all analyzed polymorphisms were in HWE and allele frequencies were comparable to those reported in the dbSNP database for Caucasian populations.

We selected mainly functional variants in order to assess their effects on breast cancer risk. However, observed associations may have arisen from other unknown functional variants in linkage with the analyzed polymorphisms in our study. In contrast, the haplotype analysis was exploratory because functionality of the analyzed haplotypes is unknown. Therefore, further studies with respect to functionality of haplotypes are necessary.

Limitations due to the retrospective case-control design are of less importance when assessing genetic variations, and thus selection bias is unlikely to have biased our results. However, when interpretating the null interactions with serum 25(OH)D, the low response rate in the population controls and measurement of 25(OH)D after diagnosis in the cases may be of concern. Information on 25(OH)D status, diet, or vitamin D related variables such as outdoor activity in nonparticipants was not available. A cancer diagnosis may change dietary or behavioural habits, which may influence 25(OH)D concentrations. Modification of dietary habits after a cancer diagnosis appears to be limited [42], but cases might have had less opportunity for outdoor activities (sun exposure) after diagnosis, leading to potential differences in 25(OH)D status between cases and controls. The median difference between time of diagnosis and time of blood collection in the cases was, however, fairly low (median [25th to 75th percentile] = 80 [14 to 260] days). The null results for the interaction between genotypes/haplotypes and 25(OH)D might also have been biased by potential influence of chemotherapy on 25(OH)D concentration. However, a notable change in 25(OH)D concentration after chemotherapeutic treatment was not observed in two studies [43,44].

Our findings are not representative for non-Caucasian populations because women in our population are primarily Caucasian and allele frequencies vary widely among populations of different ethnic origin.

## Conclusion

None of the analyzed polymorphisms was associated with risk for breast cancer overall. However, the t allele of the TaqI polymorphism was associated with a significantly increased breast cancer risk in ER-positive tumours only. In a haplotype analysis the haplotype FtCA (FokI F, TaqI t, *VDR*-5132 C, Cdx2 A) was associated with a significantly higher breast cancer risk as compared with the most frequent haplotype (FTCG). No significant interaction between *VDR *SNPs or haplotypes and serum 25(OH)D was found. Our results support potential effects of *VDR *polymorphisms on postmenopausal breast cancer risk. Further epidemiological studies assessing the association of vitamin D and breast cancer risk should take the receptor status of the tumour and other gene variants of oestrogen metabolism into account. In addition, more studies on certain polymorphisms and haplotypes in the *VDR*, especially functional studies with respect to impact on VDR activity and concentration, are needed.

## Abbreviations

1,25(OH)_2_D = 1,25-dihydroxyvitamin D; 25(OH)D = 25-hydroxyvitamin D; CI = confidence interval; ER = oestrogen receptor; HWE = Hardy-Weinberg equilibrium; NHS = Nurses Health Study; OR = odds ratio; PCR = polymerase chain reaction; PR = progesterone receptor; R-N-K = Rhein-Neckar-Karlsruhe; SNP = single nucleotide polymorphism; VDR = vitamin D receptor.

## Competing interests

The authors declare that they have no competing interests.

## Authors' contributions

SA performed the laboratory analysis and data analysis, and drafted the manuscript. AN contributed to the genotyping analysis and revised the manuscript. JL gave advice on the data analysis and contributed to writing of the manuscript. TS, SK and EJM reviewed the manuscript and participated in designing the study. DF and JC were responsible for the study design, gave advice on the data analysis and revised the manuscript. All authors read and approved the final manuscript.
